# Laser cluster interaction in ambient magnetic fields for accelerating electrons in two stages without external injection

**DOI:** 10.1038/s41598-022-14816-4

**Published:** 2022-07-04

**Authors:** Kalyani Swain, Sagar Sekhar Mahalik, Mrityunjay Kundu

**Affiliations:** 1 Institute for Plasma Research, Bhat, Gandhinagar, 382 428 India; 2 Homi Bhabha National Institute, Training School Complex, Anushaktinagar, Mumbai, 400094 India

**Keywords:** Astrophysical plasmas, Laser-produced plasmas, Plasma-based accelerators, High-harmonic generation, Atomic and molecular physics, Lasers, LEDs and light sources

## Abstract

In the few-cycle pulse regime of laser-cluster interaction (intensity $>10^{16}\,\text{ W/cm}^{2}$, wavelength $> 780$ nm), laser absorption is mostly collisionless and may happen via anharmonic resonance (AHR) process in the overdense (cluster) plasma potential. Many experiments, theory and simulation show average absorbed energy per cluster-electron (${\mathcal {E}_A}$) close to the electron’s ponderomotive energy ($U_\mathrm {p}$) in the collisionless regime. In this work, by simple rigid sphere model (RSM) and detailed particle-in-cell (PIC) simulation, we show enhanced ${\mathcal {E}_A}\approx$ 30–70$U_\mathrm {p}$—a 15–30 fold increase—with an external (crossed) magnetic field near the electron-cyclotron resonance (ECR). Due to relativistic mass increase, electrons quickly deviate from the standard (non-relativistic) ECR, but time-dependent relativistic-ECR (RECR) happens which also contributes to enhanced ${\mathcal {E}_A}$. Here laser is coupled to electrons in two stages, i.e, AHR and ECR/RECR. To probe further we retrieve the phase-difference $\Delta \psi$ between the driving electric field and corresponding velocity component for each electron (in PIC and RSM). We find absorption by electron via AHR happens in a *very short* interval $\Delta \tau$ for less than half a laser period where $\Delta \psi$ remains close to $\pi$ (necessary condition for maximum laser absorption) and then $\Delta \psi$ drops to its initial $\pi /2$ (meaning no absorption) after such *short-lived* AHR. On the contrary, auxiliary magnetic field near the ECR modifies AHR scenario inside the cluster and also helps maintaining the required phase $\Delta \psi \approx \pi$ for the liberated cluster-electron accompanied by frequency matching for ECR/RECR for a prolonged $\Delta \tau$ (which covers 50–60% of the laser pulse through pulse maxima) even after AHR—leading to jump in ${\mathcal {E}_A}\approx$ 30–70$U_\mathrm {p}$. We note that to realize the second stage of enhanced energy coupling via ECR/RECR, the first stage via AHR is necessary.

## Introduction

Intense laser-matter interaction generates energetic charge particles and photons on efficient coupling of laser^1^. Experiments show that atomic-clusters, a nanometric form of matter possessing solid-like atom-density locally in a carrier gas^2^, absorb more than 80% of laser compared to laser-solid and laser-atomic gas^3^ interaction. Initially laser field (above a critical strength) ionizes individual atoms of the cluster (called inner ionization) and forms nano-plasma. Subsequently, many electrons leave the cluster by absorbing laser energy (called outer ionization) resulting charge non-neutrality and electrostatic field transiently. Synergetic action of laser and induced electric field may create even higher charge states for multi-electron atom cluster. This process (called ionization ignition^4–7^) saturates eventually since the restoring force of ions prevents outer ionization. Simultaneously, bare ionic background expands due to ion-ion coulomb repulsion and electrostatic field energy is converted to ion kinetic energy. Experimentally detected energetic ions^3,8–10^, neutrals^11^, electrons^8,12–15^ and x-rays^16–20^ are the outcomes of this efficient laser-cluster interaction (LCI).

Clearly, laser is first coupled to the cluster-electrons and then other secondary processes begin. Laser absorption via electron-ion collision (CA) is insignificant for laser intensity $I_0>10^{16}\,\text{ W/cm}^{2}$ and wavelength $\lambda > 600$ nm^21–24^. In this regime, right after the inner ionization, increasing ion charge density $\rho _\mathrm {i}(t)$ causes the Mie-plasma frequency $\omega _\mathrm {M}(t)\!=\!\sqrt{{4\pi \rho _\mathrm {i}(t)}/{3}}$ to overtake the laser frequency $\omega = 2\pi c/\lambda$ and plasma becomes overdense (atomic units (a.u.) $\vert e\vert = m_0 = \hslash = 4\pi \epsilon _0 = 1$ are used unless noted explicitly). Later, $\rho _\mathrm {i}(t)$ gradually drops due to Coulomb expansion and the famous linear resonance (LR) condition $\omega _\mathrm {M}(t)\!=\!\omega$ is met during the laser pulse, typically after tens of femtosecond. Absorption via LR has been widely studied^25–28^. It is possible to create higher charges and more laser is absorbed by LR as the electric field enhancement dominates shielding inside the cluster. However, in the few-cycle pulse-regime $\sim 10$-fs or below^29,30^, insufficient cluster-expansion forbids LR. But, as the driven electron’s excursion amplitude *r*(*t*) increases beyond the harmonic regime of the over-dense potential by the laser, its eigen-frequency $\Omega [r(t)]$ drops below $\omega _\mathrm {M}$. Anharmonic resonance (AHR) occurs in the anharmonic potential when decreasing $\Omega [r(t)]$ of electron meets $\omega$ and the electron is promptly ejected out of the cluster with irreversible energy gain^31–34^. AHR was shown as a strong collisionless mechanism with short pulses by rigid sphere models (RSM), molecular dynamics (MD) and particle-in-cell (PIC) simulations^31–37^. So called “vacuum heating”^38,39^ for LCI is less clear for $I_0<\!\!10^{18}\,\text{ W/cm}^{2}$.

While many experiments demonstrated energetic electrons^12–15,17,40^ with $I_0<10^{18}\,\text{ W/cm}^{2}$ for $\lambda \approx 780-800$ nm, and various mechanisms were proposed through analytical models^8,24,31–34,37,41^ and numerical simulations^8,42–44^ to justify experimental findings; *still* there is no consensus for the maximum energy ${\mathcal {E}_A}^{max}$ that an electron can gain (on an average) for a given set of laser and cluster parameters. In fact, historic experiment^13^ claiming multi-keV electron energy and its double peak spectrum were later called into question^14,40^; but values of ${\mathcal {E}_A}^{max}$ in those cases were found about $\approx 2.2$ times the electron’s ponderomotive energy $U_\mathrm {p}= I_0/4\omega ^2$ (the average energy of a free electron in an oscillating field $\sim \sqrt{I_0}\sin {\omega t}$). Similarly, various oscillator models^24,31–34,37,41^, MD and PIC simulations^33,34,37,41,42,45–49^ showed ${\mathcal {E}_A}^{max}$ near $3.2U_\mathrm {p}$. Thus our extensive survey (see Table 1) reveals that in the collisionless regime of LCI, value of ${\mathcal {E}_A}^{max}$ *mostly* remains close to the famous $3.17 U_\mathrm {p}$ of the laser-atom interaction^50–52^; except in a few cases^43,44,53^ where electron’s energy around $8U_\mathrm {p}$ (or more) were also reported which is imprecise to us. Possibly, collisional events were much active therein. Nevertheless, *the primary objective* of this work is to increase ${\mathcal {E}_A}^{max}$ of cluster-electron *far beyond* $U_\mathrm {p}$.

We concentrate in the fascinating 5-fs (fwhm) short-pulse regime of laser ($I_0 >10^{15}\,\text{ W/cm}^{2}$, $\lambda = 800$ nm) interacting with a small cluster $\approx 3$ nm where both CA and LR can be ignored and AHR is applicable. By RSM and detailed PIC simulations here, AHR *alone* is shown to yield ${\mathcal {E}_A}^{max}\lesssim 3.2U_\mathrm {p}$ similar to earlier works^31–34,37,41^. We retrieve the phase-difference $\Delta \psi$ between the driving laser electric field and corresponding velocity component for each electron (in PIC and RSM) in the laser polarization; and find that *fast* generation of electrons via AHR occurs within a *tiny interval* $\Delta \tau$ where $\Delta \psi$ remains close to $\pi$ (necessary condition for maximum energy absorption rate). This condition $\Delta \psi \approx \pi$ holds only for $\Delta \tau$ less than half a laser period $T=2\pi /\omega$ and then $\Delta \psi$ quickly drops to its initial $\pi /2$ (meaning no further absorption) after such *short-lived* AHR. Though remaining laser pulse has adequate supply of energy to the AHR-freed electron, it can’t retain finally to conserve the canonical momentum. Therefore, coupling of this *unused* laser energy to the AHR-freed electron requires a *second mechanism* which is envisaged here with an ambient magnetic field $\varvec{B}_{ext}$; the electron may be energized meeting the electron-cyclotron resonance (ECR) when its cyclotron frequency $\Omega _\mathrm {c0} = \vert e\varvec{B}_{ext}/m_0 \vert = \omega$. Such ECR requires a facility for strong $B_{ext} \approx$ 10–20 kT for 800 nm laser. For $\mathrm {CO_2}$ laser (wavelength $\approx 10.6\, \mu \mathrm {m}$), the requirement of ambient magnetic field strength for ECR is lowered and $B_{ext} \approx$ 1–2 kT is sufficient which seems to be feasible. Self-generated (quasi-static) magnetic field of tens of kilo-Tesla are also often measured in the background of laser-plasma experiments and astro-physical conditions. For example self-generated magnetic fields in the range of 20–46 kT have been measured almost two decades ago in laser plasma experiments^54,55^. Magnetic field strengths in the environment of neutron stars and pulsars exceed 10 kT^56^ and typically lie in the range of $\approx$ 10–100,000 kT. Understanding of the origin of energetic electrons in these strong electromagnetic field conditions are of fundamental interest and the present work may explain them. Recent laboratory demonstration of pulsed magnetic fields from sub kilo-Tesla^57,58^ and kilo-Tesla to mega-Tesla^59–63^ has already renewed interest in laser-plasma^64–66^ community and may serve our purpose. From the practical application point of view, energetic electrons produced by LCI via ECR in the presence of an ambient magnetic field can be helpful for the table-top intense radiation sources (such as x-rays) and particle-accelerators which are useful for medical applications. Energetic electrons may help accelerating plasma ions and neutrals^11^ via secondary process (e.g., charge exchange, recombination etc.) which are also useful in medical applications and material science.

We show an enhanced ${\mathcal {E}_A}^{max}\approx$ 30–70$U_\mathrm {p}$, almost 15–30 fold increase, with an ambient $\varvec{B}_{ext}$ (in crossed orientation) near the ECR even with non-relativistic $I_0 \sim 10^{15}-2\times {10}^{17}\text{ W/cm}^{2}$. Due to relativistic mass increase with increasing energy ($\gamma m_0 c^2$), electrons may quickly deviate from the standard (non-relativistic) ECR condition $\Omega _\mathrm {c0} = \omega$, but time-dependent relativistic-ECR (RECR) happens with relativistic electron-cyclotron frequency $\Omega _c = \Omega _\mathrm {c0}/\gamma (t) = \omega$ during the laser pulse driving. The ambient $\varvec{B}_{ext}$ near the ECR not only modifies AHR scenario inside the cluster, it helps maintaining the required phase $\Delta \psi \approx \pi$ as well as frequency matching for ECR/RECR for the liberated electron from the cluster in the free space. This phase matching $\Delta \psi \approx \pi$ is maintained for a prolonged duration—$\Delta \tau$ extending 50–60% of the 5-fs broadband pulse—through pulse maxima even after the AHR, leading to huge absorption ${\mathcal {E}_A}^{max}\approx$ 30–70$U_\mathrm {p}$. Here AHR first sets a transverse momentum with which liberated electron is self-injected (no external injection scheme is required) into the remaining laser field where $\varvec{B}_{ext}$ re-orients its momentum and helps energizing it further in the free-space enforcing improved phase-matching $\Delta \psi \approx \pi$ and frequency matching for ECR/RECR. However, to realize the ECR/RECR stage (second stage), a transverse momentum of the electron through AHR (first stage) is necessary. Our PIC results are shown to be well-supported by RSM.

## Methods

### Rigid sphere model of field driven cluster

In the RSM, cluster is assumed as a pre-ionized spherical nano-plasma of radius *R* and fixed ionic charge density $\rho _\mathrm {i}$. RSM has been widely used for LCI^29–34,37,67^ without $\varvec{B}_{ext}$. In this work we first include $\varvec{B}_{ext}$ in RSM to study its effects. Ions provide the potential $\phi (r)$ with the space-charge field

1 $$\varvec{E}_{sc} (\varvec{r}) = \left\{ {\begin{array}{*{20}l} \omega _\mathrm {M}^{2} \varvec{r} \hfill & \text {if }r \le R \hfill \\ \omega _\mathrm {M}^{2} R^3 \varvec{r}/{r^3} \hfill & \text {if }r > R \hfill \\ \end{array} } \right.$$

in which electrons interact in addition to the applied laser field ($\varvec{E}_l, \varvec{B}_l$) and external $\varvec{B}_{ext}$. Dynamics of an electron obeys

2 $$\begin{aligned} \frac{d\varvec{p}}{dt}&= q\left[ \left( \varvec{E}_l + \varvec{E}_{sc}(\varvec{r}) \right) + \varvec{v}\times \left( \varvec{B}_l + \varvec{B}_{ext} \right) \right] \end{aligned}$$

3 $$\begin{aligned} \frac{d\varvec{r}}{dt}&= \varvec{v} = \frac{\varvec{p}}{\gamma m_0} \end{aligned}$$

4 $$\begin{aligned} \frac{d({\gamma } m_{0} c^2) }{dt}&= q {\varvec{v}}.\left( \varvec{E}_l + \varvec{E}_{sc}(\varvec{r}) \right) \end{aligned}$$

where $\gamma = 1/\sqrt{1-v^2/c^2}= \sqrt{1+p^2/m_{0}^2 c^2}$ is the relativistic $\gamma$-factor for the electron, $m_{0}, q, \varvec{r}, \varvec{v}, \varvec{p}$ are its rest-mass, charge, position, velocity and linear momentum respectively with $m_0 = 1$, $q = e = -1$ in a.u. Equations (1)–(3) represent a field driven three-dimensional non-linear oscillator. The coulomb part of $\varvec{E}_{sc} \propto \varvec{r}/{r^3}$ restricts its analytical solution, except in some simplified linear case of $\varvec{E}_{sc} \propto \varvec{r}$ with continuous (plane-wave) laser field only. For example, see direct laser acceleration (DLA) of electrons from an under-dense, pre-formed plasma channel^65,68–72^ assisted by auxiliary fields, e.g., magnetic wigglers, static electric and magnetic fields with $I_0 >10^{18}\,\text{ W/cm}^{2}$ and corresponding normalized vector potential $a_0 = \sqrt{I_0}/\omega c > 1$. To obtain electrons of MeV energies (or higher), the regime of $a_0 > 1$ is an obvious choice. Such pre-formed plasma channels are very long (typically tens of $\lambda$) and relativistically intense laser has to propagate several $\lambda$ which then sets up electro-static fields in the channel with associated self-generated quasi-static magnetic fields. Electrons are injected into the channel or drawn from the plasma itself and guided by the channel’s fields and the applied laser field. If the ambient magnetic field is in the direction transverse to the laser polarization, then energy of electrons can be increased and ECR may happen if such magnetic field satisfies the ECR condition. This work, however, reports other unexplored regime of DLA with $I_0 < 10^{18}\,\text{ W/cm}^{2}$ using short-pulsed light and a constant $\varvec{B}_{ext}$ for an over-dense cluster plasma electrons.

The field $\varvec{E}_{sc}$ imparts oscillatory motion in $\varvec{r}$, whereas $\varvec{B}_{ext}$ imparts rotation in the plane perpendicular to $\varvec{B}_{ext}$ (in $\varvec{r}_{\perp }$) to an electron. Combining these two motions, the position dependent squared effective-frequency ${\omega }_{\mathrm {eff}}^2[\varvec{r}(t)]$ of electron in the RSM [using (1)–(2)] can be formally obtained as

5 $$\begin{aligned} {\omega }_{\mathrm {eff}}^2[\varvec{r}(t)] = \varvec{\hat{r}} \cdot (\gamma \varvec{E}_{sc}/ r + \Omega _{c0}^2 \varvec{\hat{r}_{\perp }})/\gamma ^2. \end{aligned}$$

The term $(\gamma \varvec{E}_{sc}/r + \Omega _{c0}^2 \varvec{\hat{r}_{\perp }}) \cdot \varvec{\hat{r}_{\perp }}/\gamma ^2$ represents motion due to combined space-charge and $\varvec{v}\times \varvec{B}_{ext}$ field in $\varvec{r}_{\perp }$ plane and $(\gamma \varvec{E}_{sc} \cdot \varvec{\hat{r}_{||}})/\gamma ^2 r$ represents motion in $\varvec{\hat{r}_{||}}$ along $\varvec{B}_{ext}$. The unit vectors $\varvec{\hat{r}}, \varvec{\hat{r}_{\perp }}$ indicate frequencies are valid only for motions in those directions. Equation (5) may be regarded as the relativistic extension to its non-relativistic variant^33,34,37,67,73^ for $\Omega _{c0} = 0$ and $\gamma = 1$. When $\varvec{E}_{sc} = \omega _\mathrm {M}^2\varvec{r}$ and $\gamma \approx 1$, it gives harmonic oscillator frequency ${\omega }_{\mathrm {eff}}^2 [\varvec{r}(t)] \approx (\omega _\mathrm {M}^2 + \Omega _{c0}^2)$ for low $\vert \varvec{B}_{ext}\vert$ values inside the cluster where $\omega _\mathrm {M}\gg \Omega _{c0}$, $\varvec{r}_{||}\approx 0$. It may also be looked upon as upper-hybrid electron frequency^74,75^ in magnetized plasmas. Due to non-linear $\varvec{E}_{sc}$ and the relativistic non-linearity imposed by strong $\varvec{E}_l, \varvec{B}_l, \varvec{B}_{ext}$; the ${\omega }_{\mathrm {eff}}^2 [\varvec{r}(t)]$ drops from ${\omega }_{\mathrm {eff}}^2 [0] \approx (\omega _\mathrm {M}^2 + \Omega _{c0}^2)$ for increasing $r> R$. An electron may absorb laser energy by AHR when its ${\omega }_{\mathrm {eff}}[\varvec{r}(t)]$ dynamically meets the condition ${\omega }_{\mathrm {eff}}[\varvec{r}(t)]=\omega$ for increasing $r> R$ above a certain strength of $\vert \varvec{E}_l\vert$. Significance of AHR was explained^31–37,41^. Here, we shall also present modified AHR (using Eq. (5)) with $\varvec{B}_{ext}$.

*The laser pulse.* We assume a laser pulse^41,67^ of vector potential $\varvec{A}_l(t') = \hat{\mathbf {x}} ({E_0}/{\omega })\sin ^2({\omega t'}/{2n}) \cos (\omega t')$ for $0 \le t' \le nT$ which is polarized in *x* and propagating in *z*; where $t' = t - {z}/c$, $n=$ number of period *T*, $\tau = n T$, and $E_0=\sqrt{I_0}$. The $\varvec{E}_l$, $\varvec{B}_l$ read

6 $$\begin{aligned} \varvec{E}_l (t')&= \hat{\mathbf {x}} \frac{E_0}{\omega } \!\!{\left\{ \begin{array}{ll} \sum _{i=1}^{3}c_i\omega _i\sin (\omega _i t') &{}\!\!\!\quad\text {if }0 \le t' \le nT \\ 0 &{}\text {otherwise}; \end{array}\right. } \end{aligned}$$

7 $$\begin{aligned} \varvec{B}_l (t')&= \hat{\mathbf {z}} \times \varvec{E}_l (t') / c \end{aligned}$$

where $c_1=1/2, c_2=c_3= -1/4, \omega _1 = \omega , \omega _2 = (1+1/n)\omega$, and $\omega _3 = (1-1/n)\omega$. For $R\ll \lambda$, the dipole approximation $z/\lambda \ll 1$ may be assumed.

*The cluster.* A deuterium cluster with number of atoms $N=2176$ and $R\approx 2.05$ nm is irradiated by above laser pulse for $n=5$, $\tau = nT\approx 13.5$ fs ($\tau _{fwhm}\sim 5$ fs), unless explicitly mentioned. Cluster is $\rho _i/\rho _\mathrm {c}\approx 27.1$ times overdense with $(\omega _\mathrm {M}/\omega )^2 \approx 9.1$, where $\rho _\mathrm {c}\approx 1.75\times 10^{27} m^{-3}$ is the critical density at 800 nm. Equations (1)–(3) using Eqs. (6)–(7) are numerically solved by the Velocity Verlet method (VVM).

#### Regeneration of previous RSM results: single electron dynamics with laser field only

**Figure 1:**
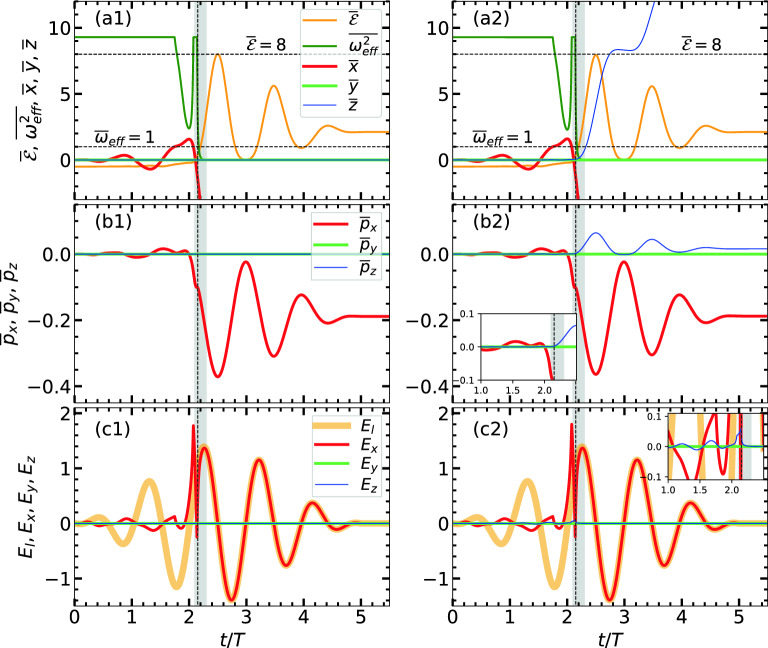
Conventional RSM results without $\varvec{B}_{ext}$: showing (column-wise) normalized ($\overline{x}, \overline{y}, \overline{z}$), $\overline{\omega }_{\mathrm {eff}}^2$, $\overline{\mathcal {E}}$ and ($\overline{p}_x, \overline{p}_y, \overline{p}_z$) vs *t*/*T* of an initially bound electron $[\varvec{r}(0)=\varvec{0}, \varvec{p}(0)=\varvec{0}]$ in the RSM potential when driven by $n=5$-cycle pulse of $I_0 \approx 7.13\times 10^{16}\,\text{ W/cm}^{2}$. Associated $E_x = \varvec{\hat{x}}.(\varvec{E}_l + \varvec{E}_{sc}), E_y = \varvec{\hat{y}}.\varvec{E}_{sc}$, $E_z = \varvec{\hat{z}}.\varvec{E}_{sc}$ and $E_l = \varvec{\hat{x}}.\varvec{E}_l$ vs *t*/*T* are also plotted. Panels in the left (**a1,b1,c1**) and right (**a2,b2,c2**) columns represent the cases with $\varvec{B}_l = 0$ and $\varvec{B}_l \ne 0$. The deuterium cluster has number of atoms $N=2176$, radius $R\approx 2.05$ nm giving $\overline{\omega }_{\mathrm {eff}}^2[r(0)] = \omega _\mathrm {M}^2/\omega ^2 \approx 9.1$. AHR and outer-ionization occur at $t/T\approx 2.1$ (vertical dashed line, shaded bar) in both cases. $\varvec{B}_l \ne 0$ imparts a forward momentum and excursion in *z*. Inset plots (in **b2,c2**) show zoomed view of momenta and fields inside the cluster.

We begin with energy absorption and associated electron’s dynamical variables as a conventional case of LCI without $\varvec{B}_{ext}$. Figure 1 shows (column-wise) normalized co-ordinates ($\overline{x}= x/R, \overline{y}= y/R, \overline{z}= z/R$), squared effective frequency $\overline{\omega }_{\mathrm {eff}}^2={\omega }_{\mathrm {eff}}^2/\omega ^2$, total energy $\overline{\mathcal {E}} = ((\gamma -1)m_0 c^2 + q\phi )/U_\mathrm {p}$ in units of $U_\mathrm {p}$ and corresponding momenta ($\overline{p}_x= p_x/c, \overline{p}_y= p_y/c, \overline{p}_z= p_z/c$) vs time *t*/*T* of an initially bound electron $[\,\varvec{r}(0)=\varvec{0}, \varvec{p}(0)=\varvec{0}\,]$ in the RSM potential, when driven by 5-cycle pulse of $I_0 = 7.13\times 10^{16}\,\text{ W/cm}^{2}$. Associated fields $E_x = \varvec{\hat{x}}.(\varvec{E}_l + \varvec{E}_{sc}), E_y = \varvec{\hat{y}}.\varvec{E}_{sc}$, $E_z = \varvec{\hat{z}}.\varvec{E}_{sc}$ and $E_l = \varvec{\hat{x}}.\varvec{E}_l$ vs *t*/*T* are also plotted, purpose of which will be evident when we consider $\varvec{B}_{ext}$ later. Left panels (a1,b1,c1) and right panels (a2,b2,c2) are the cases with $\varvec{B}_l = \varvec{0}$ and $\varvec{B}_l \ne \varvec{0}$ respectively.

Without $\varvec{B}_l$ (in many works $\varvec{B}_l$ was neglected^21,27,31,76^ since $\max \vert \varvec{B}_l\vert =E_0/c \ll 1$), it is shown that electron starts (Fig. 1a1) with the binding energy $\overline{\mathcal {E}} = -1.5\omega _\mathrm {M}^2 R^2/U_\mathrm {p}$ at $t/T=0$, it oscillates in the potential with increasing amplitude in *x* (while $y=0,z=0$) as the total field ${E}_x$ oscillates in time (while $E_y = 0, E_z=0$) and approaches to the peak value $E_0$ of $\varvec{E}_l$ (Fig. 1c1) around $t/T\approx 2$. Inside the potential, for $r/R\le 1$, $E_x$ is suppressed due to opposite phase of $\varvec{\hat{x}}.\varvec{E}_l$ and $\varvec{\hat{x}}.{\varvec{E}_{sc}}$. As long as $r/R\le 1$, $\overline{\omega }_{\mathrm {eff}}^2$ continues (Fig. 1a1) at its initial value $\omega _\mathrm {M}^2/\omega ^2 \approx 9.1$. When ${E}_x$ increases sufficiently strong (due to reduced phase mismatch between $\varvec{\hat{x}}.\varvec{E}_l$ and $\varvec{\hat{x}}.{\varvec{E}_{sc}}$) leading to increasing $r/R>1$; $\overline{\omega }_{\mathrm {eff}}^2$ falls rapidly, it meets the AHR condition $\overline{\omega }_{\mathrm {eff}}^2 = 1$ around $t/T\approx 2.1$ (marked by horizontal dashed line and vertical shaded bar) and then electron leaves the cluster forever (Fig. 1a1) with $\overline{\mathcal {E}}>0$ associated with non-zero transverse momentum $\overline{p}_x$ (in Fig. 1b1). After the AHR, $\varvec{E}_x$ follows $\varvec{E}_l$. Though LR can not happen, AHR is dynamically met here leading to the electron’s removal from the cluster with $\overline{\mathcal {E}}>0$ and non-zero $\overline{p}_x$ eventually. Similar results (neglecting $\varvec{B}_l$) are shown in Refs.^31–34,37,41^.

Considering $\varvec{B}_l$ now, Fig. 1a2,b2,c2 show indistinguishable variation of ($\overline{x},\overline{y},\overline{\omega }_{\mathrm {eff}}^2, \overline{\mathcal {E}}, \overline{p}_x, \overline{p}_y, E_x, E_y$) with respective Fig. 1a1,b1,c1. Also $\overline{z}\approx 0, \overline{p}_z\approx 0$ (in a2, b2) before the occurrence of AHR near $t/T\approx 2.1$, since $\varvec{v}\times \varvec{B}_l$ field along *z* is much weaker and leads to a negligible $E_z$ [Fig. 1c2, clearly seen in zoomed inset plots in (b2,c2)]. As the electron is liberated (Fig. 1a2) via AHR around $t/T\approx 2.1$ with dominant velocity in *x* (Fig. 1b2), the $\varvec{v}\times \varvec{B}_l$ field imparts a forward momentum $p_z$ along the laser propagation (Fig. 1b2) and its *z* co-ordinate sharply increases (Fig. 1a2) by many times *R*. Electron is now emitted in the $z-x$ plane with an angle $\theta \approx \arctan (p_x/p_z)$ in contrast to Fig. 1a1,b1 where electron is emitted *only* along the polarization axis. Though $I_0 < 10^{18}\,\text{ W/cm}^{2}$, the liberated electron via the AHR process around $t/T\approx 2.1$ is *self-injected* into the remaining laser pulse with some forward momentum $\overline{p}_z>0$ and transverse momentum $\overline{p}_x$; and from this time onward electron’s acceleration resembles the standard DLA. Clearly, inclusion of $\varvec{B}_l$ here yields (Fig. 1a2,b2,c2) *different electron dynamics* (see also Mulser et al.^31^) for LCI than neglecting it^21,27,31,41,76–78^ in previous works.

However, both the cases in Fig. 1 show maximum attainable energy $\max {\overline{\mathcal {E}}} = 8$ (marked by upper horizontal dashed line) near the laser peak at $t/T= 2.5$; but the electron retains only a lower value of energy $\overline{\mathcal {E}}_A = \overline{\mathcal {E}}(\tau ) \approx 2.1$ in the end. We may compare these two limits of $\overline{\mathcal {E}}$ with the laser-driven electron-atom re-collision model^50–52,79,80^ of harmonic generation where $\max {\overline{\mathcal {E}}}$ of electron may go up to $\approx 8$ during the pulse, but the returned electron when re-collides with the parent ion has a lower $\overline{\mathcal {E}}\approx 3.17$ which is often manifested as a harmonic cut-off energy. In laser-cluster experiments, an electron’s final energy is reported to be less than the above mentioned laser-atom interaction case^81–83^ and the final absorbed energy limit $\overline{\mathcal {E}}_{A}^{max} = \max {\overline{\mathcal {E}}_A} \lesssim 3.17$ seems to obey^8,14,40^ herein. Particle simulations^33,34,37,41,42,45–49,67^ and simple models^24,31–34,37,41,67^ employed so far for LCI also indicate $\overline{\mathcal {E}}_{A}^{max} \lesssim 3.17$ in the collision-less case. Thus, though the role of $\varvec{B}_l$ can not be neglected for altering the electron dynamics (Fig.1a2,b2,c2) at a $I_0>7.13\times 10^{16} \text{ W/cm}^{2}$ (where peak magnetic field can be substantial $> 2.44$ kT), the average $\overline{\mathcal {E}}_{A}^{max} \lesssim 3.17$ seems to follow (see Table 1) for the traditional LCI. The aim of the paper is to increase this limit far beyond $\overline{\mathcal {E}}^{max}_{A} \sim 3.17$ with an ambient $\varvec{B}_{ext}$.

### PIC simulation

We also study LCI with/without $\varvec{B}_{ext}$ using three-dimensional PIC simulation code^33,34,41,76,78,84,85^. The same deuterium cluster with number of atoms $N = 2176$ is considered. Atoms are placed in a cubical computational box according to the Wigner-Seitz radius $r_w\approx 0.17$ nm (giving cluster radius $R=r_w N^{1/3} \approx 2.05$ nm) so that center of the cluster coincides the center of the computational box of side $L = 24.6 R$. Initially laser $\varvec{E}_l(t)$ ionizes all neutral atoms D to D$^{+}$ (assuming over-the-barrier ionization, OBI^86^ which is valid for $I_0>10^{15}\,\text{ W/cm}^{2}$) after reaching a critical strength $E_c = \vert \varvec{E}_l(t)\vert = I_p^2(Z)/4 Z$, where $I_p(Z)$ is the ionization potential for charge state $Z=1$. Such a fully ionized cluster initially acquires a charge density $\rho _\mathrm {i}/\rho _\mathrm {c}\approx 27.87$ and $\omega _\mathrm {M}/\omega \approx 3.05$ at 800 nm. Thus cluster parameters are kept as the RSM. The position and velocity of a newly born electron (after the OBI) are assumed same as the parent atom/ion conserving the momentum and energy. Subsequent movement of more mobile electrons from the relatively less mobile ions by the driving fields create/modify space-charge field $\varvec{E}_{sc}(\varvec{r},t)$. Thus $\varvec{E}_{sc}(\varvec{r},t) = -\varvec{\nabla }\phi (\varvec{r},t)$ and corresponding potential $\phi (\varvec{r},t)$ in PIC are time-dependent and start from zero contrary to the RSM.

A PIC electron/ion has the same charge to mass ratio of a real electron/ion. The equation of motion of the $j\vert k$-th PIC electron/ion (*j* for electron and *k* for ion) reads

8 $$\begin{aligned} \frac{d\varvec{p}_{j\vert k}}{dt}\! = q_{j\vert k}\! \left[ \left( \varvec{E}_l(t) + \varvec{E}_{sc}(\varvec{r}_{j\vert k},t) \right) + \varvec{v}_{j\vert k}\times \left( \varvec{B}_l + \varvec{B}_{ext} \right) \right] \end{aligned}$$

where $\varvec{p}_{j \vert k} = m_{j\vert k} \varvec{v}_{j\vert k}/\sqrt{1-v_{j\vert k}^2/c^2}, \varvec{v}_{j\vert k}, \varvec{r}_{j \vert k}, m_{j\vert k}, q_{j\vert k}$ are relativistic momentum, velocity, position, mass, and charge of a PIC electron/ion respectively. In the present case, $m_j = m_0 = 1$, $m_k = M_0 = 2\times 1386$, $q_j = -1$ and $q_k = 1$ in a.u.. Poisson’s equation $\nabla ^2\phi _G = -\rho _G$ is solved for $\phi _G$ on the numerical grid (subscript *G* indicates grid values of potential and charge density) with time-dependent monopole boundary condition. Interpolating $\phi _G$ to the particle position corresponding potential $\phi (\varvec{r}_{j\vert k},t)$ is obtained. Field $\varvec{E}_{sc}(\varvec{r}_{j\vert k}) = -\varvec{\nabla } \phi (\varvec{r}_{j\vert k})$ in (8) is obtained by analytical differentiation^76^ of interpolated $\phi (\varvec{r}_{j\vert k})$ locally at $\varvec{r}_{j\vert k}$. Equation (8) is solved by VVM using laser fields (6)–(7). Total absorbed energy $\mathcal {E}(t) = \sum _l q_l\phi _l + p_l^2/2 m_l$ is obtained by summing over all electrons and ions. For the 5-fs pulse (used here) contribution of ion kinetic energy is small and total energy is mainly due to electrons. The numerical parameters in the PIC simulation (spatial and temporal resolution, grid size, number of PIC particles/cell etc.) are carefully chosen for negligible artificial numerical heating. Typically, we have chosen $64\times 64\times 64$ grid points (cells) with uniform grid size $\Delta x=\Delta y = \Delta z = 16$ a.u., time step $\Delta t = 0.1$ a.u., and approximately 15 particles/cell. Two important upgrades are made in the current PIC version: relativistic particle mover based on (i) Runge-Kutta 4-th order method (RK4) and (ii) VVM. It is found that VVM leads to better energy conservation and less numerical heating even for a bigger $\Delta t$ than RK4, particularly for the relativistically intense driving fields. Electron-ion collisions are neglected in the current work due to high field values.

## Results

### New RSM results: electron dynamics with laser and auxiliary $B_{ext}$

**Figure 2:**
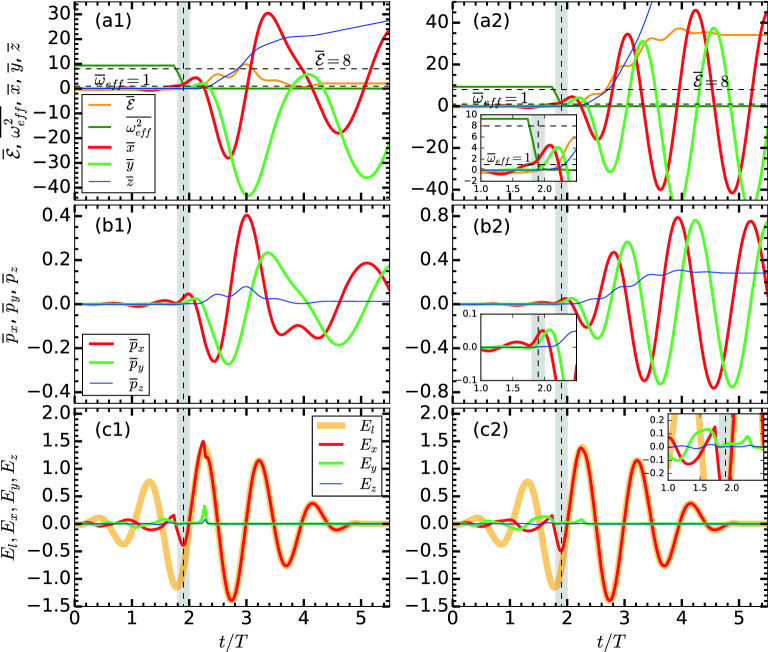
Modified RSM results with $(\varvec{E}_{l},\varvec{B}_{l})$ and $\varvec{B}_{ext}$ along *z*: showing dynamical variables of the same electron as in Fig. 1. Panels in the left (**a1,b1,c1**) and right (**a2,b2,c2**) columns are with ${B}_{ext} \approx 0.028, 0.0569$ a.u. corresponding to non-resonant $\Omega _{c0}/\omega = 0.5$ and resonant $\Omega _{c0}/\omega = 1$ (ECR case) respectively. In the ECR case AHR occurs little early around $t/T \approx 1.85$, and $\overline{\mathcal {E}}_A$ reaches up to $\overline{\mathcal {E}}(\tau )\approx 36$ compared to $\overline{\mathcal {E}}_A=\overline{\mathcal {E}}(\tau )\approx 2.1$ in Fig. 1a2; corresponding momenta and excursion also significantly vary after the electron is freed via AHR near $t/T \approx 1.85$. Inset plots (in **a2,b2,c2**) show zoomed view of dynamical variables near AHR and inside the cluster due to strong $\varvec{B}_{ext}$. Other laser and cluster parameters are as in Fig. 1.

Results in Fig. 1 (right column) show that for the chosen $\varvec{E}_l,\varvec{B}_l$ configuration, a liberated electron from cluster may also gain a mild forward momentum $\overline{p}_z$ after AHR. The energy-momentum relation $\overline{p}_z- \overline{p}_{z0}= (\gamma -\gamma _0)c$ for DLA (without space-charge) suggests that to improve energy gain by the electron, its $\overline{p}_z$ should be increased from the initial $\overline{p}_{z0} = \gamma _0 c$. Though magnetic field does not work, an auxiliary $\varvec{B}_{ext}$ helps bending electron’s trajectory. It may also improve $\overline{p}_z$ of the freed electron.

Figure 2a1,b1,c1 show results with ${B}_{ext}=\vert \varvec{\hat{z}} {B}_{ext}\vert \approx 6.68$ kT (corresponding $\Omega _{c0}= \omega /2$) along *z* for the same $(\varvec{E}_l,\varvec{B}_l)$ as in Fig. 1a2,b2,c2 which is considered as a reference. Noticeably, variation of dynamical variables are now very different from the corresponding Fig. 1a2,b2,c2; but the final retained energy of the electron is still $\overline{\mathcal {E}}_A \approx 2.1$. The $\overline{\omega }_{\mathrm {eff}}^2$ starts at $\approx (\omega _\mathrm {M}^2 + \Omega _{c0}^2)/\omega ^2$, monotonically drops and passes the AHR line $\overline{\omega }_{\mathrm {eff}}^2=1$ (horizontal dashed line) at a little early time $t/T\approx 1.85$ (vertical shaded bar) following Eq. (5) contrary to its short-time oscillatory nature just before the occurrence of AHR (Fig. 1a2) near $t/T\approx 2.1$. The vanishing of oscillatory nature of $\overline{\omega }_{\mathrm {eff}}^2$ (Fig. 2a1) and its smooth passage through the $\overline{\omega }_{\mathrm {eff}}^2=1$ line is due to additional induced fields $E_y, E_z$ [though still weak, Fig. 2c1] due to strong $\varvec{B}_{ext} = \hat{z} {B}_{ext}$ leading to swirling motion in (*x*, *y*) inside the cluster similar to the driving by a circularly polarized laser field^33,73^. Thus an external $\varvec{B}_{ext} = \hat{z} {B}_{ext}$ may modify electron dynamics inside the cluster and the AHR scenario. The ($\overline{x}, \overline{y}, \overline{p}_x, \overline{p}_y$) dynamics of the liberated electron tends to follow cyclotron motion; both $\overline{p}_z$ and $\overline{\mathcal {E}}$ grow up-to a maximum (note that $\max {\overline{\mathcal {E}}}\approx 10.5$ now exceeds the conventional ${\overline{\mathcal {E}}}=8$ line without $\varvec{B}_{ext}$) near the pulse peak. But $\overline{p}_z$ drops later (Fig. 2b1) leading to lesser (Fig. 2a1) final energy $\overline{\mathcal {E}}_A = \overline{\mathcal {E}}(\tau )\approx 2.1$ as in Fig. 1a2 though electron dynamics drastically differ from Fig. 1a2,b2,c2.

With a higher ${B}_{ext} = \vert \varvec{\hat{z}} {B}_{ext}\vert \approx 13.37$ kT corresponding to $\Omega _{c0} = \omega$ (ECR), Fig. 2a2,b2,c2 show a *significant jump* in the final absorbed energy upto $\overline{\mathcal {E}}_A\approx 36$ (far exceeding the conventional $\overline{\mathcal {E}}_A^{max} \approx 3.17$) associated with a jump in the corresponding final $\overline{p}_z\approx 0.3$. Most of the arguments relevant to Fig. 2a1,b1,c1 apply here also. Additional inset plots are zoomed view of dynamical variables near AHR and inside the cluster. Due to higher $\varvec{B}_{ext} = \varvec{\hat{z}} B_{ext}$, induced fields $E_y, E_z$ in the cluster (Fig. 2c2) are also marginally stronger, AHR scenario is marginally modified and note that, even in this case $\overline{p}_z,\overline{z}$ are almost zero inside the cluster. Distinctly, after the AHR near $t/T\approx 1.85$, liberated electron follows almost exact cyclotron motion in the $x-y$ plane (evident from $\overline{x}, \overline{y}, \overline{p}_x, \overline{p}_y$ variation) due to the stronger $\varvec{B}_{ext} = \varvec{\hat{z}} B_{ext}$, while its $\overline{p}_z$ and $\overline{\mathcal {E}}$ continuously increase to $\overline{p}_z\approx 0.3$ and $\overline{\mathcal {E}}\approx 36$ during $t/T\approx 2-4$ followed by saturation, though laser field envelope (Fig. 2c2) weakens after its peak. Thus an external magnetic field -assisted electron acceleration from a laser-driven cluster is shown to enhance electron’s energy by 10–12 times than the conventional limit of $\overline{\mathcal {E}}_A^{max} \approx 3.17$, particularly near the ECR frequency $\Omega _{c0}= \omega$. This encouraging *new result* needs further investigation.

Above results with ${B}_{ext}$ show laser absorption happens mainly in two stages. In the first stage, electron undergoes AHR (may be modified by ${B}_{ext}$) and comes out of the cluster with low positive energy and non-zero transverse momentum. Later, in the second stage, it is fully controlled by the remaining $\varvec{E}_l,\varvec{B}_l$ and $\varvec{B}_{ext}$ with an increase in $\overline{\mathcal {E}}$, i.e., absorption rate (see Fig. 2a2) for $t/T\approx 2-4$. This second stage may be termed as magnetic field assisted DLA. However, to realize the second stage, energy absorption by electron and its liberation from the cluster in the first stage *is necessary*, otherwise $\varvec{v}\times \varvec{B}_{ext}$ fails.

**Figure 3:**
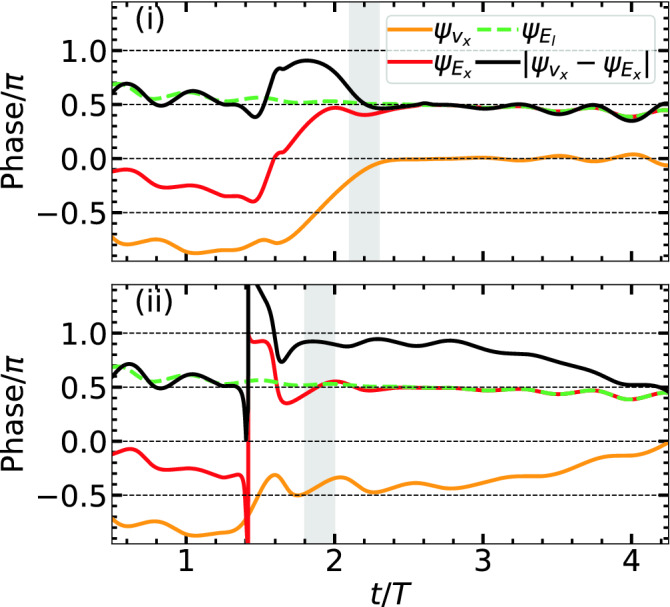
Temporal variation of phase angles $\psi _{v_x}, \psi _{E_x}, \psi _{E_l}$ of respective $v_x, E_x, E_l$ associated with the same RSM electron (i) in Fig. 1a2,b2,c2 without $\varvec{B}_{ext}$ and (ii) in Fig. 2a2,b2,c2 with ${B}_{ext} = \vert \hat{z} \varvec{B}_{ext}\vert \approx 0.0569$ (ECR case). Phases are numerically calculated by FFT w.r.t. the central frequency $\omega$ with a sliding time-window duration $T=2\pi /\omega$. In (i) relative phase $\Delta \psi = \vert \psi _{v_x}-\psi _{E_x}\vert$, on an average, stays close to $\pi$ (or $0.75\pi$) for a short-while $\Delta \tau <T/2$. In (ii) $\Delta \psi$ stays close to $\pi$ for a prolonged duration $\Delta \tau > T$ through the pulse peak (before falls to $\pi /2$ at $t/T\approx 4$) leading to $\overline{\mathcal {E}}_A \approx 36$ in Fig. 2a2.

*The temporal phase dynamics.* Equation (4) implies that rate of absorption ${d{\gamma } m_{0} c^2 }/{dt}$ by an electron approaches to zero (or negligible) for phase angle $\Delta \psi$ between its velocity and the corresponding driving electric field approaching to the odd integral multiple of $\pi /2$. From Eq. (4), one may apparently conclude no role of $\varvec{B}_{ext}$ for enhanced absorption in Fig. 2a2,b2,c2. Note that in the second stage of energy absorption, where role of ${\varvec{E}_\mathrm {sc}}$ is nil, Eq. (4) simplifies to ${d{\gamma } m_{0} c^2 }/{dt} = q {v_x} {E}_x = q v_x E_l$; and, though $\varvec{B}_{ext}$ can not alter $E_l$, it may re-orient the phase of $v_x$ (see the cyclotron orbit) w.r.t. $E_x$. To probe *this underlying physics*, we numerically retrieve phase angles $\psi _{v_x}, \psi _{E_x}, \psi _{E_l}$ w.r.t. central frequency $\omega$ of respective $v_x, E_x, E_l$ (since components along the laser polarization matter the most) for two cases: (i) with $\varvec{E}_l,\varvec{B}_l$ only for Fig. 1a2,b2,c2 and (ii) with $\varvec{E}_l,\varvec{B}_l$ and ${B}_{ext} \approx 13.37$ kT for Fig. 2a2,b2,c2. Those $\psi _{v_x}, \psi _{E_x}, \psi _{E_l}$ and $\Delta \psi = \vert \psi _{v_x}-\psi _{E_x}\vert$ vs time are plotted in Fig. 3 (see caption). Vertical shaded bars are the respective AHR regions (see Figs. 1, 2) after which the electron is mostly free from space-charge fields of the cluster and respective $\psi _{E_x}$ goes hand in hand with $\psi _{E_l}$ in both cases. Little deviation of $\psi _{E_l}$ from $\pi /2$ far away from the pulse center (at $t/T=2.5$) is due to 5-cycle broad-band pulse (ideally it is $\pi /2$ for a monochromatic pulse $\sim \sin \omega t$). Respective $\psi _{v_x}, \psi _{E_x}$ in (i) do not differ from (ii) and $\Delta \psi \approx 0.5\pi$ remains upto $t/T\approx 1.4$. After this time, $\psi _{v_x}, \psi _{E_x}$ in (i) increase slowly for $t/T\approx 1.4-1.75$ where $\Delta \psi \approx 0.5\pi \rightarrow \pi$ [$\Delta \psi \approx 0.9\pi$ is maintained for a tiny duration $\Delta \tau$] followed by its gradual drop through the AHR region and saturation near $\Delta \psi \approx 0.5\pi$ afterwards. On the contrary, in (ii) an instantaneous phase swing occurs (near $t/T\approx 1.4$) for $\psi _{E_x} = -\pi \rightarrow \pi$ after quick dropping to $-\pi$. Later, though $\psi _{E_x} \rightarrow \psi _{E_l} \approx 0.5 \pi$, the phase $\psi _{v_x}$ is dynamically tilted in a way that a value of $\Delta \psi \approx \pi$ is brought about by the auxiliary ${B}_{ext}$ for a long duration $t/T\approx 1.75-3.0$ (leading to high absorption rate in Fig. 2a2) from pre-AHR to post-AHR time through the pulse maxima; then $\Delta \psi$ gradually drops as $\approx \pi \rightarrow 0.5\pi$ for $t/T\approx 3.0-4.0$ where absorption slows down and finally saturates at a higher $\overline{\mathcal {E}}_A\approx 36$ in Fig. 2a2. Thus an auxiliary ${B}_{ext}$ near the ECR helps maintaining the required $\Delta \psi \approx \pi$ for enhanced laser absorption in the second stage.

#### RSM results: absorption with different orientation of ${B}_{ext}$

**Figure 4:**
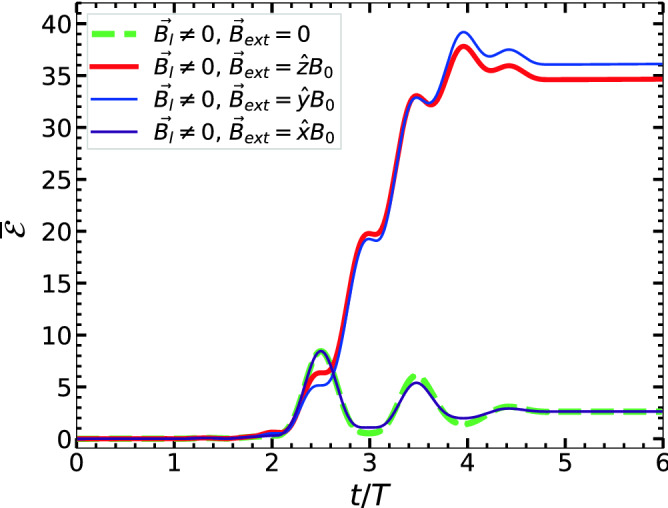
RSM results: Time vs absorbed energy in units of $U_\mathrm {p}$ of the single electron (in Fig. 2) for different $\varvec{B}_{ext}$: (i) $\varvec{B}_{ext} = \varvec{0}$, (ii) $\varvec{B}_{ext} = \varvec{\hat{z}} {B}_{ext}$, (iii) $\varvec{B}_{ext} = \varvec{\hat{y}} {B}_{ext}$ and (iv) $\varvec{B}_{ext} = \varvec{\hat{x}} {B}_{ext}$. Laser fields ($\varvec{E}_l,\varvec{B}_l$) and magnitude of $\vert \varvec{B}_{ext}\vert \approx 13.37$ kT are as in Fig. 2a2,b2,c2. Cases (ii) and (iii) are only two energetically favorable orientations of $\varvec{B}_{ext}$.

For further understanding of magnetic field-assisted laser-energy coupling we study similar ECR cases with same conditions of Fig. 2a2,b2,c2 but other orientations of $\varvec{B}_{ext}$. For the sake of conciseness, we plot energy vs time in Fig. 4 for: (i) $\varvec{B}_{ext} = \varvec{0}$, (ii) $\varvec{B}_{ext} = \varvec{\hat{z}} {B}_{ext}$, (iii) $\varvec{B}_{ext} = \varvec{\hat{y}} {B}_{ext}$ and (iv) $\varvec{B}_{ext} = \varvec{\hat{x}} {B}_{ext}$ where ${B}_{ext} \approx 13.37$ kT. Results show almost same level of enhanced absorption upto $\overline{\mathcal {E}}_A = \overline{\mathcal {E}}(\tau )\approx 35-36$ only when $\varvec{B}_{ext}\perp \varvec{E}_l$ [cases (ii) and (iii)], although electron dynamics are different here. When $\varvec{B}_{ext} || \varvec{E}_l$, there is no enhancement in the final energy [case (iv)] and gives the same level of $\overline{\mathcal {E}}_A \approx 2.1$ as in the case (i) since $\varvec{v}\times \varvec{B}_{ext} \approx \varvec{0}$. Thus RSM quickly identifies possible directions of $\varvec{B}_{ext}$ for enhanced laser absorption. Now onwards we focus on the results with $\varvec{B}_{ext} = \varvec{\hat{z}} {B}_{ext}$ only.

#### Non-interacting multi-electrons in RSM

A single-electron dynamics (as studied by RSM above) is important to understand the physics of LCI, but can not answer some other aspects, e.g., fraction of electrons leaving the cluster (outer ionization fraction) and their energy distribution. In the single-electron case, outer-ionization fraction assumes only 0,1 (electron is either inside or outside the cluster). In a real system, however, some electrons may remain bound and outer-ionization fraction may attain any value between (0,1) depending upon laser and cluster parameters. A single-electron case may over-estimate/under-estimate electron energy compared to the realistic multi-electron case where per-electron energy may be averaged out. Moreover, different electrons become free from the cluster at different times, and participate in the magnetic field assisted DLA differently. To answer these aspects we distribute all $N=2176$ electrons inside the cluster randomly (or uniformly) to mimic a multi-electron system by RSM where electrons are assumed non-interacting among them. For brevity, we compare these multi-electron results of RSM along with detailed PIC simulation in the following section where particle-particle interactions are taken care self-consistently.

### Absorption studies with PIC simulation and comparison with RSM

**Figure 5:**
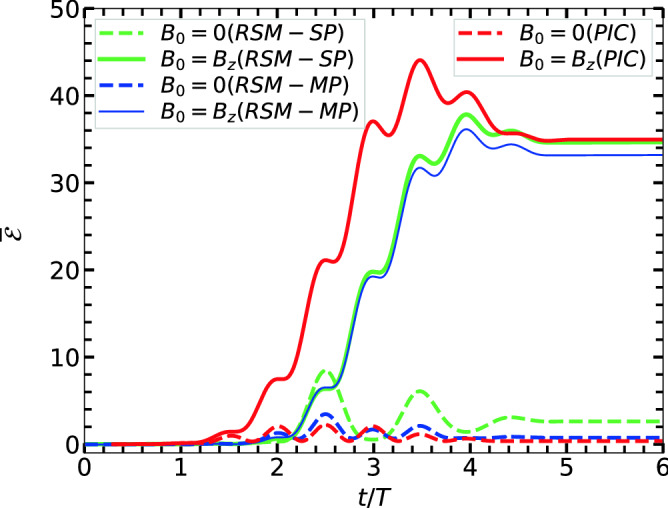
Comparison of PIC and RSM results: average total absorbed energy $\overline{\mathcal {E}}(t) = {\mathcal {E}}(t)/NU_\mathrm {p}$ per particle in $U_\mathrm {p}$ vs *t*/*T* with $B_{ext}=0$ (dashed lines, conventional case of Fig. 1a2,b2,c2) and with $B_{ext} = \omega$ (solid lines, ECR case of Fig. 2a2,b2,c2). RSM results with single-electron (RSM-SP) and non-interacting multi-electrons (RSM-MP) both justify PIC results. Though absorption starts early in PIC, final absorbed energies $\overline{\mathcal {E}}_A = \overline{\mathcal {E}}(\tau )$ with/without $B_{ext}$ are comparable with the RSM cases.

Figure 5 compares time vs average energy (per electron) between PIC and RSM results for $B_{ext} = 0$ and $\vert \varvec{\hat{z}} B_{ext}\vert = \omega$ (ECR case) at $I_0\approx 7.13\times 10^{16}\text{ W/cm}^{2}$. RSM results with single-electron (RSM-SP) as in Fig. 4 and non-interacting multi-electrons (RSM-MP) as described in section above are also included. RSM-SP over-estimates the RSM-MP case for final energy $\overline{\mathcal {E}}_A = \overline{\mathcal {E}}(\tau )$ when $B_{ext} = 0$, but PIC result ($\overline{\mathcal {E}}_A \approx 0.5$) follows RSM-MP more closely. For the ECR case, however, $\overline{\mathcal {E}}_A \approx 36$ in PIC remains little higher than RSM-MP, which is due to early ejection of electrons with non-zero transverse momentum via AHR from the self-consistently developing potential and electro-static restoring fields (starting from zero) in PIC. Note that $\overline{\mathcal {E}}(t)$ starts increasing one-period earlier ($t/T\approx 1.2$) in PIC than the RSM and so as the ECR for those early leaving PIC electrons. Almost $60-70$ fold increase in $\overline{\mathcal {E}}_A\approx 0.5 \rightarrow 36$ is obtained in PIC and RSM-MP due to $\vert \varvec{\hat{z}} B_{ext}\vert$ near ECR.

**Figure 6:**
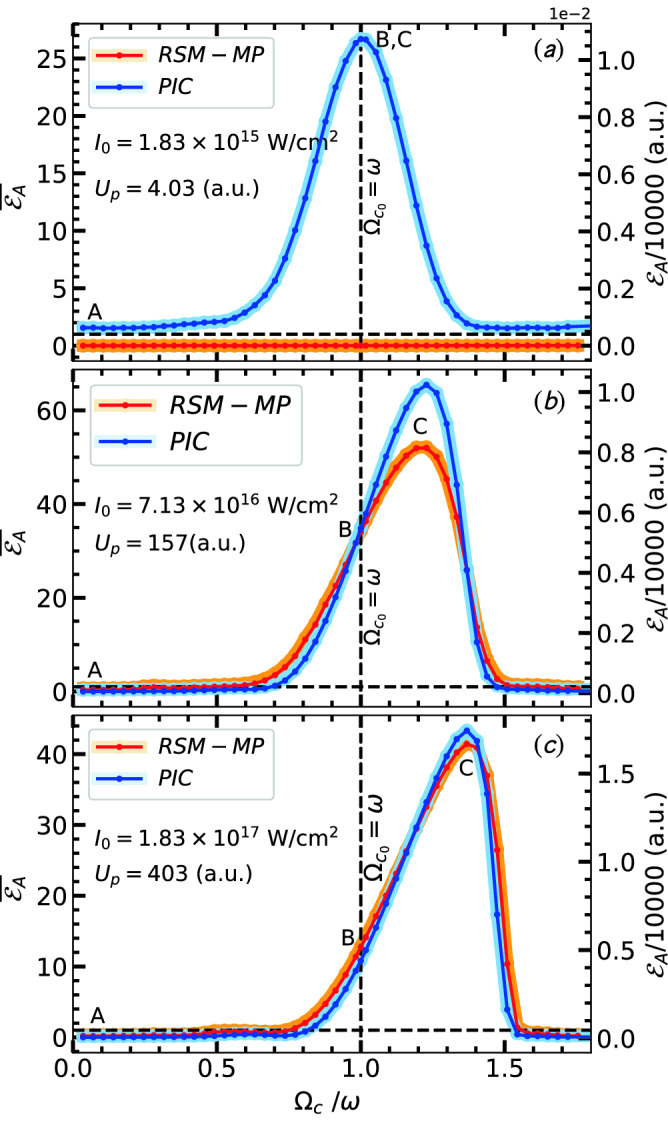
Comparison of PIC and RSM results: Average absorbed energy $\overline{\mathcal {E}}_A=\overline{\mathcal {E}}(\tau )$ per particle vs $\Omega _{c0}/\omega$ for a range of $\vert \varvec{\hat{z}} B_{ext}\vert \approx (0 - 2\omega )$ with $n=5$-cycle pulses of different $I_0\approx 1.83\times 10^{15}\text{ W/cm}^{2}- 1.83\times 10^{17}\text{ W/cm}^{2}$. Energy is shown normalized by corresponding $U_\mathrm {p}$ (left y-axis) and in atomic units (right y-axis). At a low intensity (**a**) absorption peaks almost at the ECR condition $\Omega _{c0} = \omega$ (vertical dashed line) as clearly exhibited by PIC where electrons undergo AHR at ease and become free with transverse momentum for the ECR in the next stage; whereas RSM-MP shows almost zero absorption since AHR is not met (first stage fails) in RSM. As $I_0$ increases to moderate values (in **b,c**) absorption peaks show-up in RSM-MP due to meeting of AHR followed by ECR. For high intensity RSM-MP justify PIC results quantitatively. Gradual right-shift of the absorption peak from ECR condition $\Omega _{c0} = \omega$ with increasing $I_0$ is due to relativistic modification of $\Omega _{c} = \Omega _{c0}/\gamma$ for $\gamma >1$. Absorption peaks $\approx 65U_\mathrm {p}, 45U_\mathrm {p}$ in (**b,c**) give average energy per electron ${\mathcal {E}}_A\approx 0.27, 0.49$ MeV respectively.

**Figure 7:**
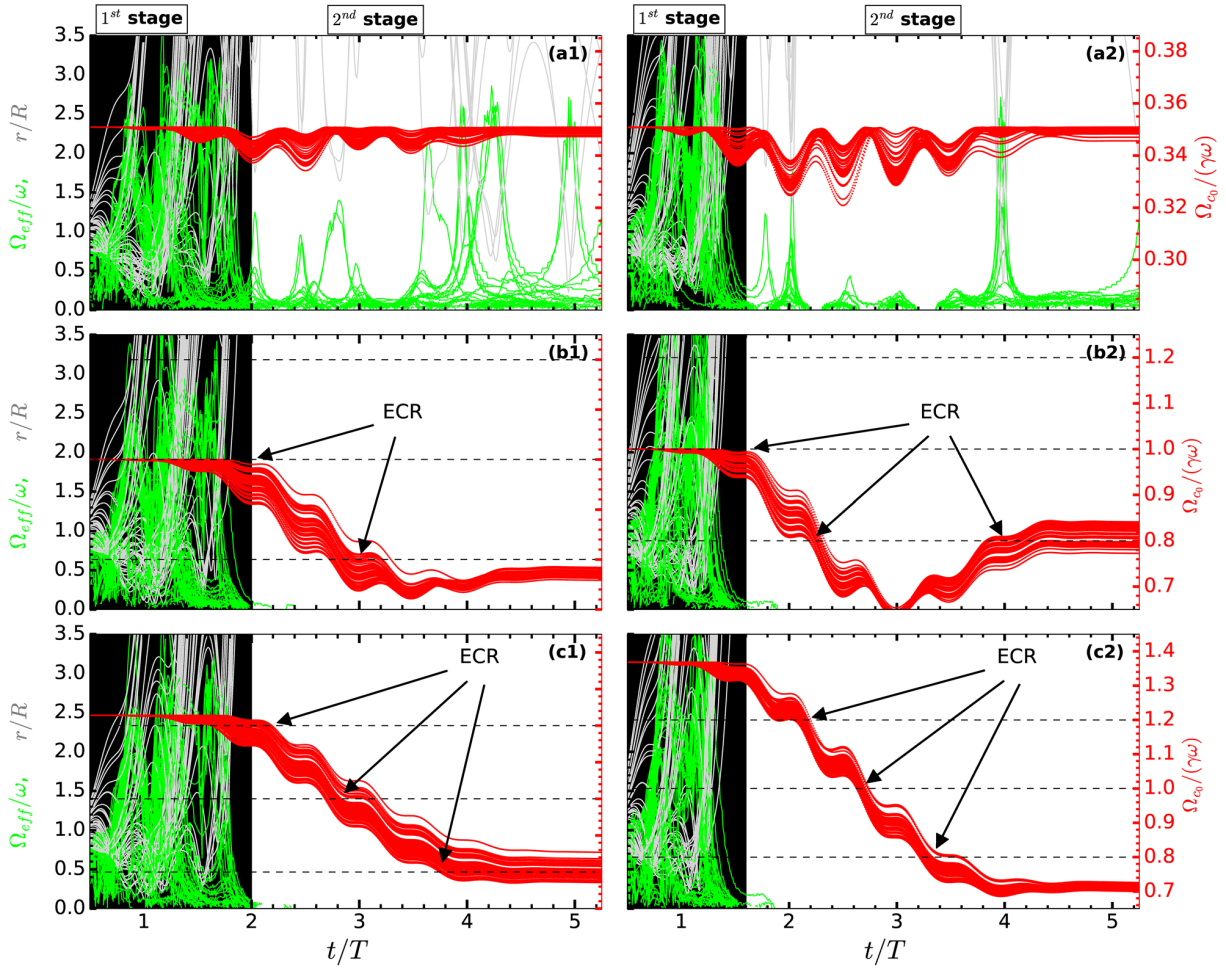
Time vs frequency analysis for PIC electrons: Normalized ${\Omega }_{\mathrm {eff}}/\omega$ (green, left y-axis) and $\Omega _{c}/\omega$ (red, right y-axis) of cluster electrons for $\vert \hat{z} \varvec{B}_{ext}\vert \approx 0.02, 0.0569, 0.07$ at $I_0 = 7.13\times 10^{16}\,\text{ W/cm}^{2}$ (left column, **a1,b1,c1**) and $\vert \hat{z} \varvec{B}_{ext}\vert \approx$ 0.02, 0.0569, 0.078 at $I_0 = 1.83\times 10^{17}\,\text{ W/cm}^{2}$ (right column, **a2,b2,c2**) corresponding to PIC results (at **A,B,C**) in Fig. 6b,c respectively. Vertical shaded region indicates AHR region where ${\Omega }_{\mathrm {eff}}/\omega$ of each electron starts from zero, reaches different maximum, then drops to zero passing through AHR when electron is freed from the cluster potential with excursion $r/R\gg 1$ (gray) and non-zero transverse momentum. Horizontal dashed lines represent frequencies of the broadband pulse where ECR/RECR are expected. At low $B_{ext}$ values, ECR is not met (**a1,a2**), laser absorption is mainly due to AHR occurring for $t/T\le 2$ (1st stage, vertical shaded region). As $B_{ext}$ increases, $\gamma$ of electrons increase, all frequencies of the broadband pulse gradually come under ECR/RECR condition (second stage) with decreasing $\Omega _{c}/\omega$ as one passes (**b1,b2**) to (**c1,c2**). In (**c1,c2**) ECR/RECR is hit around the peak of the pulse (at $t/T=2.5$) with central frequency $\omega$ as well as with side-bands $1.2\omega , 0.8\omega$ leading to higher absorption in (**c1,c2**) compared to the case (**b1,b2**). In (**b1,b2**) ECR is hit in the beginning of the pulse with $\omega$ when laser field is relatively weak, then RECR with the side-band at $0.8\omega$ near the pulse peak and in the pulse end (for **b2**). Note that ECR/RECR occurring at very early time ($t/T<1.5$) or very late time ($t/T>4$) are less effective due to weak laser field. Other laser and cluster parameters are as in Fig. 1. See also Fig. 8 for corresponding phase dynamics.

Scanning through range of values of $$\vert \varvec{\hat{z}} B_{ext}\vert \approx (0 - 2\omega )$$, for different $$I_0\approx 1.83\times 10^{15}\text{ W/cm}^{2}- 1.83\times 10^{17}\text{ W/cm}^{2}$$ and same 5-fs pulse duration, results in Fig. 6 are obtained by PIC and RSM-MP in the end of the pulses. At a low intensity (Fig. 6a) absorption peak (at $$26\,U_\mathrm {p}$$) occurs almost at the ECR condition $$\Omega _{c0} = \omega$$ (vertical dashed line) as clearly exhibited by PIC simulation where electrons can undergo AHR at ease, become free with a transverse momentum for the ECR in the next stage; whereas RSM-MP shows almost zero absorption since AHR is not met (first stage fails) and electrons can’t be freed from RSM potential with a transverse momentum at this low intensity (RSM greatly under-estimates absorption here, since its needs a threshold intensity^31,32^). Therefore, as the peak intensity increases, absorption peaks show-up gradually (Fig. 6b,c) for RSM-MP due to gradual removal of electrons via AHR (preferably) from surface to the cluster center, *but* ECR absorption peak occurs always for PIC. Finally, at a higher $$I_0\approx 1.83\times 10^{17}\text{ W/cm}^{2}$$, PIC and RSM (almost overlap) show very good quantitative agreement in Fig. 6c. Absorption peaks $$\approx 65U_\mathrm {p}, 45U_\mathrm {p}$$ in Fig. 6b,c give average energy $${\mathcal {E}}_A\approx 0.27, 0.49$$ MeV. The gradual right-shift of the absorption peak from the ECR condition $$\Omega _{c0} = \omega$$ (vertical dashed line) with increasing intensity is due to the relativistic modification of $$\Omega _c = \Omega _{c0}/\gamma$$ in dipole-approximation. Since $$\gamma$$ is time-varying (during the pulse) and different for different electrons, the time-dependent relativistic-ECR occurs for electrons when $$\Omega _c (t) = \Omega _{c0}/\gamma (t) = \omega$$ (call it RECR, see Fig. 7). It emphasizes quick slippage of electron from the RECR condition as soon as its $$\gamma (t)>1$$. Therefore, to satisfy the RECR for $$\gamma >1$$, a higher $$\Omega _{c0}$$ (or higher $$B_{ext})$$ is required—as manifested by gradual right-shift of the absorption peak (Fig. 6–c) with increasing intensity. Moreover, laser pulse being broadband with frequencies $$\omega , (1\pm 1/n)\omega$$, RECR may happen in a wider frequency range and contribute to broadening of resonance-width about the absorption peak in Fig. 6.

**Figure 8:**
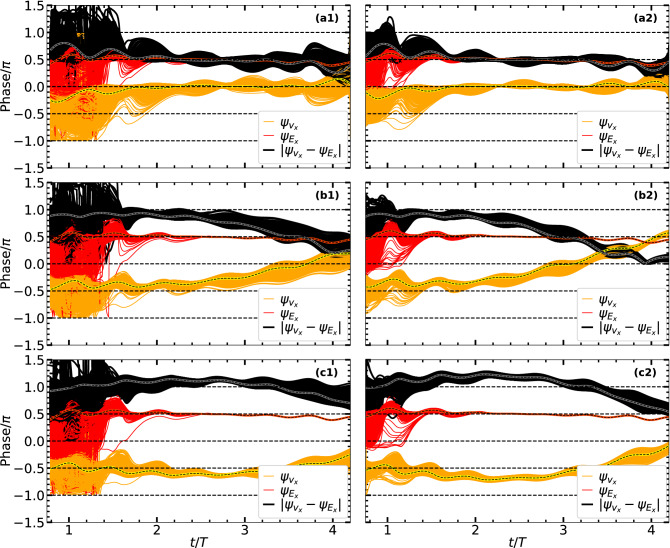
Time vs phase analysis for PIC electrons: Phase-angles $\psi _{v_x}, \psi _{E_x}$ of respective dynamical variables $v_x, E_x$ and phase difference $\Delta \psi = \vert \psi _{v_x}-\psi _{E_x}\vert$ of all $N=2176$ electrons for $\vert \hat{z} \varvec{B}_{ext}\vert \approx 0.02, 0.0569, 0.07$ at $I_0 = 7.13\times 10^{16}\,\text{ W/cm}^{2}$ (left column, **a1,b1,c1**) and $\vert \hat{z} \varvec{B}_{ext}\vert \approx 0.02, 0.0569, 0.078$ at $I_0 = 1.83\times 10^{17}\,\text{ W/cm}^{2}$ (right column, **a2,b2,c2**) corresponding to PIC results (at **A,B,C**) in Fig. 6b,c respectively. Wavy dashed lines indicate respective phase angles for average values $\sum {v_x}/N, \sum {E_x}/N$ of all *N* electrons showing average system behavior removing rapid phase fluctuations. At low $B_{ext}$ values, ECR is not met, $\Delta \psi$ quickly falls to $\pi /2$ after initial rise towards $\pi$ due to AHR (mostly occurring) for $t/T\le 2$. As $B_{ext}$ increases towards ECR, $\Delta \psi$ is gradually lifted towards $\pi$, and it is maintained for a longer duration $\Delta \tau \approx 60-70\%$ of the pulse through pulse maximum leading to higher absorption in Fig. 6b,c even after AHR. Phase angles are numerically computed by FFT as in RSM Fig. 3. See also Fig. 7 for corresponding frequency dynamics. Other laser and cluster parameters are as in Fig. 1.

*Frequency and phase dynamics of PIC electrons.* To elucidate further, we retrieve the relativistic anharmonic eigen-frequency ${\Omega }_{\mathrm {eff}}$ and cyclotron-frequency $\Omega _{c}$ for each $k-th$ PIC electron as (see also Eq. (5))

9 $$\begin{aligned} {\Omega }_{\mathrm {eff}}^2&= \varvec{\hat{r}} \cdot \varvec{E}_{sc}(r_k)/\gamma _k r_k \end{aligned}$$

10 $$\begin{aligned} \Omega _{c}^2&= \varvec{\hat{r}}\cdot (\Omega _{c0}^2 \varvec{\hat{r}_{\perp }})/\gamma _k^2. \end{aligned}$$

Figure 7 shows temporal variation of ${\Omega }_{\mathrm {eff}}/\omega$ (green, left y-axis) and $\Omega _{c}/\omega$ (red, right y-axis) of cluster electrons for $\vert \hat{z} \varvec{B}_{ext}\vert \approx 0.02, 0.0569, 0.07$ a.u. at $I_0 = 7.13\times 10^{16}\,\text{ W/cm}^{2}$ (left column, a1,b1,c1) and $\vert \hat{z} \varvec{B}_{ext}\vert \approx 0.02, 0.0569, 0.078$ a.u. at $I_0 = 1.83\times 10^{17}\,\text{ W/cm}^{2}$ (right column, a2,b2,c2) corresponding to PIC results in Fig. 6b,c respectively. Chosen values of $\vert \hat{z} \varvec{B}_{ext}\vert$ for each intensity represent data points A, B, C (at the tail, at the non-relativistic ECR condition $\Omega _{c0}=\omega$, and at the peak) on the PIC absorption curves in Fig. 6b,c. Normalized position *r*/*R* of electrons (gray) show their distances w.r.t. center of the cluster. Corresponding phase-angles $\psi _{v_x}, \psi _{E_x}$ of respective $v_x, E_x$ along the laser polarization and the phase difference $\Delta \psi = \vert \psi _{v_x}-\psi _{E_x}\vert$ for each $k-th$ PIC electron are computed by FFT (as in RSM Fig. 3) and shown in Fig. 8 for both intensities. Wavy dashed lines (Fig. 8) indicate respective phase angles for average values $\sum {v_x}/N, \sum {E_x}/N$ of all *N* electrons showing average system behavior. Contrary to the RSM, ${\Omega }_{\mathrm {eff}}/\omega$ of each PIC electron starts from zero and reaches different maximum (Fig. 7) when its *r*/*R* drops towards the potential minimum. Then ${\Omega }_{\mathrm {eff}}/\omega$ each PIC electron drops to zero passing through AHR^33,34,37^ similar to the RSM and electron is freed from the cluster potential with $r/R\gg 1$. Shaded vertical bar (in Fig. 7) highlights this AHR dominated region (1st stage) during initial time of the laser pulse. Since different electrons undergo AHR at different times and comes out with different non-zero transverse momentum, the exact extent of the 1st stage and the beginning of 2nd stage (ECR stage) with $B_{ext}$ is difficult to draw (i.e., minor overlap happens and 2nd stage starts early for early leaving electrons via AHR) with all electrons together. However, from the vanishing of ${\Omega }_{\mathrm {eff}}/\omega \rightarrow 0$ and increasing $r/R\gg 1$ it is clear that AHR domain (1st stage) is mostly limited below $t/T\approx 2$ for $I_0 = 7.13\times 10^{16}\,\text{ W/cm}^{2}$ ($t/T\approx 1.6$ for $I_0 = 1.83\times 10^{17}\,\text{ W/cm}^{2}$) and shrinks with increasing intensity.

At low $\vert \hat{z} \varvec{B}_{ext}\vert \approx 0.02$ (or without it) as in Fig. 7a1,a2, the frequency matching for ECR can not happen, the phase difference $\Delta \psi$ continues to $\pi /2$ after initial rise towards $\pi$ shown in respective Fig. 8a1,a2 due to short-lived AHR occurring below $t/T<2$. Hence absorbed energy remains low ($<3U_\mathrm {p}$) without initiating the second stage. In these cases not all electrons are freed (Fig. 7a1,a2), many of them may comeback inside the cluster later time, and may be liberated again through another AHR, e.g., see after $t/T>4$.

As $B_{ext}$ increases (see caption of Fig. 7), $\gamma$ of electrons increase, all frequencies of the broadband pulse (shown by horizontal dashed lines) gradually come under ECR/RECR condition with decreasing $\Omega _{c}/\omega$ as one passes Fig. 7b1,b2–c1,c2; accompanied by gradual lifting of $\Delta \psi$ towards $\pi$ *even after AHR* with more time elapsed near $\pi$ as in respective Fig. 8b1,b2–c1,c2. Also, as RECR is met with the central frequency $\omega$ near the pulse peak (Fig. 7) at $t/T=2.5$ and respective $\Delta \psi$ is maintained near $\pi$ for a longer duration $\Delta \tau \approx 60-70\%$ of the pulse through pulse maximum (Fig. 8), it leads to higher absorption (${\mathcal {E}}_A$) in Fig. 6c, b. Thus, not only frequency matching $\Omega _{c}/\omega = 1$ for ECR/RECR is satisfied, the required phase matching condition $\Delta \psi \approx \pi$ is also simultaneously satisfied by PIC electrons for all cases in Fig. 6 (same are checked with electrons in RSM-MP for Fig. 6, not repeated) for enhanced absorption peak about 30–70$U_\mathrm {p}$.

## Discussion and summary

We study laser-deuterium cluster interaction with short 5-fs (fwhm) laser pulses ($I_0 >10^{15}\,\text{ W/cm}^{2}$, $\lambda = 800$ nm) in presence of external magnetic field $B_{ext} \approx$ 10–20 kT using RSM and three-dimensional PIC simulations. For the standard case, without $B_{ext}$, our extensive survey on laser-cluster interaction finds that average energy per electron ${\mathcal {E}_A}$ most often remains around $3.2U_\mathrm {p}$ or less. Without $B_{ext}$, first we show that AHR *alone* may yield ${\mathcal {E}_A}\lesssim 3.2U_\mathrm {p}$ similar to earlier works^24,31–34,37,41,42,45–49,67^ even with the inclusion of the laser magnetic field $\varvec{B}_l$. We then retrieve the phase-difference $\Delta \psi$ between the driving laser electric field and corresponding velocity component for each electron (in PIC and RSM) in the laser polarization and find that generation of electrons via AHR occurs within a *short interval* $\Delta \tau$ where $\Delta \psi$ remains close to $\pi$ (necessary condition for maximum energy absorption rate) only for $\Delta \tau$ less than half a laser period. After that $\Delta \psi$ quickly drops to its initial $\pi /2$, leading no further absorption. Thus AHR is found to be very *short-lived*. Though remaining laser pulse supplies energy temporarily, the AHR-freed electron can not retain finally. Therefore, coupling of this unused laser energy to the AHR-freed electron is envisaged here through a *second mechanism* with $\varvec{B}_{ext}$, namely ECR, when electron-cyclotron frequency $\Omega _\mathrm {c0} = \vert e\varvec{B}_{ext}/m_0 \vert = \omega$.

**Table 1:** Approximate value of maximum average absorbed energy ${\mathcal {E}_A}^{max}$ (in units of ponderomotive energy $U_\mathrm {p}$) of an electron from traditional laser-cluster interaction in various published works.

References	Model/simulation/experiment	Approximate parameters	${\mathcal {E}_A}^{max}/U_\mathrm {p}$
^8^	Experiment	Xe cluster, $R = 3.2$ nm, $I_0=1.5\times 10^{16}$ W/cm$^2$, $\lambda = 780$ nm, $\tau = 150$fs	2.3–3.5
^13^	Experiment	Xe cluster, $R= 5$ nm , $I_0=1\times 10^{16}$ W/cm$^2$, $\lambda = 790$ nm, $\tau = 150$fs	3.54
^14^	Experiment	Xe cluster, $I_0=5\times 10^{16}$ W/cm$^2$, $\lambda = 800$ nm, $\tau = 50$fs	2.02
^15^	Experiment	Ar cluster, $R= 4$ nm , $I_0=1\times 10^{17}$ W/cm$^2$, $\lambda = 820$ nm, $\tau = 28$fs	16.03
^24^	Semi-classical model	Xe cluster, $I_0=3.51\times 10^{15}$ W/cm$^2$, $\lambda = 800$ nm, $\tau = 42$fs	2.21
^31,32^	Model	$R=10$ nm, $I_0=6.8\times 10^{17}$ W/cm$^2$, $\lambda = 800$ nm, $\tau = 30-40 fs$	2.5
^33,34^	Model and Simulation	Xe cluster, $R= 3.2$ nm, $I_0=2.5\times 10^{16}$ W/cm$^2$, $\lambda = 1056$ nm, $\tau = 28$fs	1.0–2.0
^37,41^	Model and Simulation	D cluster, $R= 2.05$ nm , $I_0=5\times 10^{15}$ W/cm$^2$, $\lambda$ = 800 nm, $\tau$= 13.5fs	1.5–2.4
^45^	Simulation	Ar cluster, $R= 3$ nm , $I_0=8\times 10^{16}$ W/cm$^2$, $\lambda$ = 806 nm, $\tau$= 70fs	1.0
^43^	Simulation	Xe cluster, $R= 5$ nm, $I_0=1\times 10^{16}$ W/cm$^2$, $\lambda$ = 800 nm, $\tau$= 400fs	7.6
^42^	Simulation	Xe cluster, $R= 2$ nm, $I_0=1\times 10^{17}$ W/cm$^2$, $\lambda$ = 248 nm, $\tau$= 8.27fs	2.1
^46^	Simulation	Xe cluster, $R= 1$ nm $-2$ nm, $I_0=1\times 10^{16}$ W/cm$^2$, $\lambda$ = 800 nm, $\tau$= 400fs	1.13–2.03
^53^	Simulation	Xe cluster, $R= 5$ nm, $I_0=1\times 10^{16}$ W/cm$^2$, $\lambda$ = 800 nm, $\tau$= 80fs	8.5
^47,48^	Simulation	Ar cluster, $R= 1.8$ nm, $I_0=2\times 10^{15}$ W/cm$^2$, $\lambda$ = 800 nm, $\tau$= 80fs	1.0
^49^	Simulation	Ar cluster, $R= 5$ nm, $I_0=1\times 10^{15}$ W/cm$^2$, $\lambda$ = 800 nm, $\tau = 25$fs	1.11
^44^	Simulation	Ar cluster, $R =$ 38 nm, $I_0=5\times 10^{15}$ W/cm$^2$ , $\lambda$ = 800 nm, $\tau$= 100fs	10.34
^83^	Review paper	Xe cluster, $R =$ 4.3 nm, $I_0=1\times 10^{15}$ W/cm$^2$, $\lambda = 800$ nm, $\tau = 250$fs	2.54

These are calculated from the available data/graphs. In most cases ${\mathcal {E}_A}^{max}$ remains close to $3.2U_\mathrm {p}$ or below.

We show an enhanced average energy per electron ${\mathcal {E}_A}\approx$ 30–70$U_\mathrm {p}$ with an ambient $\varvec{B}_{ext}$ (in crossed orientation) near the ECR even with non-relativistic $I_0 \sim 10^{15}-2\times {10}^{17}\text{ W/cm}^{2}$. Due to relativistic mass increase with increasing kinetic energy ($\gamma m_0 c^2$), electrons quickly deviate from the standard (non-relativistic) ECR condition $\Omega _\mathrm {c0} = \omega$, but time-dependent relativistic-ECR (RECR) happens with relativistic electron-cyclotron frequency $\Omega _c = \Omega _\mathrm {c0}/\gamma (t) = \omega$ during the laser pulse driving. The ambient $\varvec{B}_{ext}$ near the ECR not only modifies AHR scenario inside the cluster, it also helps maintaining the required phase $\Delta \psi \approx \pi$ as well as frequency matching for ECR/RECR for the liberated electron from the cluster in the free space for a prolonged duration $\Delta \tau$. We find that $\Delta \tau$ extends $\approx$ 50–60% of the 5-fs broadband pulse – through pulse maxima even after the AHR—leading to huge absorption ${\mathcal {E}_A}\approx$ 30–70$U_\mathrm {p}$. Here AHR first sets a transverse momentum with which liberated electron is self-injected (no external injection scheme is required) into the remaining laser field where $\varvec{B}_{ext}$ re-orients its momentum and helps energizing it further in the free-space enforcing improved phase-matching $\Delta \psi \approx \pi$ and frequency matching for ECR/RECR. This work may ignite new interest in laser-cluster interaction for energetic electron generation.

## Data Availability

The data that support the plots and findings of this paper are available from the corresponding author on reasonable request. However, due to other novel findings, authors won’t be able to make the raw data public.
